# Within outlying mean indexes: refining the OMI analysis for the realized niche decomposition

**DOI:** 10.7717/peerj.3364

**Published:** 2017-05-23

**Authors:** Stéphane Karasiewicz, Sylvain Dolédec, Sébastien Lefebvre

**Affiliations:** 1 UMR 8187 LOG (Laboratoire d’Océanologie et Géosciences), CNRS, Université des Sciences et Technologies de Lille (Lille I), ULCO, Wimereux, Nord-Pas-de-Calais, France; 2 UMR 5023 LEHNA (Laboratoire d’Ecologie des Hydrosystèmes Naturels et Anthropisés), Biodiversité et Plasticité dans les Hydrosystèmes, Université Claude Bernard (Lyon I), Villeurbanne, Rhône, France; 3 Laboratoire Ressources Halieutiques, IFREMER, Boulogne-sur-mer, Nord-Pas-de-Calais, France

**Keywords:** Biological constraint, Niche dynamic, Marginality, Community, Spatio-temporal, Subniche, Habitat

## Abstract

The ecological niche concept has regained interest under environmental change (e.g., climate change, eutrophication, and habitat destruction), especially to study the impacts on niche shift and conservatism. Here, we propose the within outlying mean indexes (WitOMI), which refine the outlying mean index (OMI) analysis by using its properties in combination with the *K*-select analysis species marginality decomposition. The purpose is to decompose the ecological niche into subniches associated with the experimental design, i.e., taking into account temporal and/or spatial subsets. WitOMI emphasize the habitat conditions that contribute (1) to the definition of species’ niches using all available conditions and, at the same time, (2) to the delineation of species’ subniches according to given subsets of dates or sites. The latter aspect allows addressing niche dynamics by highlighting the influence of atypical habitat conditions on species at a given time and/or space. Then, (3) the biological constraint exerted on the species subniche becomes observable within Euclidean space as the difference between the existing fundamental subniche and the realized subniche. We illustrate the decomposition of published OMI analyses, using spatial and temporal examples. The species assemblage’s subniches are comparable to the same environmental gradient, producing a more accurate and precise description of the assemblage niche distribution under environmental change. The WitOMI calculations are available in the open-access R package “subniche.”

## Introduction

The ecological niche concept has been reactivated due to increasing concern over global environmental change, making the niche shift and the conservatism between different areas and time periods important fields of study (Peterson, 2011). The ecological niche of a species can be decomposed into two related components (Hutchinson, 1957). First, the fundamental niche is the *n*-dimensional hypervolume within which the population of a species can persist, survive, and reproduce indefinitely, and it is not constrained by any biological interactions. Second, the realized niche is the proportion of the fundamental niche within which the species actually persist, i.e., taking into account the effect of abiotic and biological interactions. The fundamental niche cannot be measured by observation, but rather by broad examination of species’ physiological requirements using mechanistic approaches (Peterson et al., 2011). On the contrary, the realized niche, in a community context, is the “differential habitat preferences of species” (Ter Braak & Verdonschot, 1995) and can be estimated by correlative approaches (Peterson et al., 2011).

**Figure 1:**
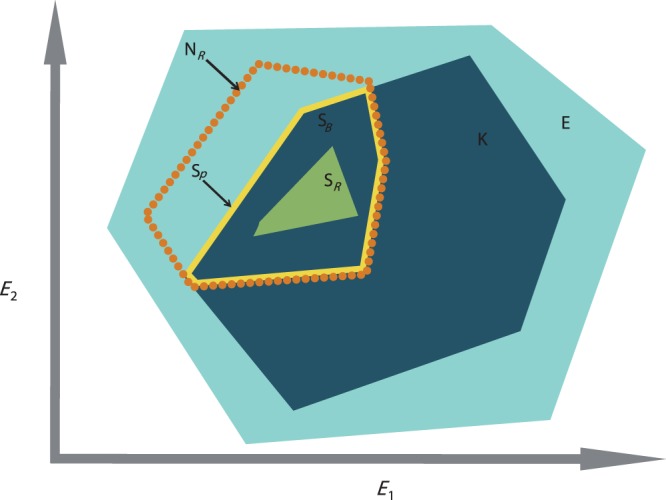
The concept of the existing fundamental niche and biotic interactions of Jackson & Overpeck (2000) adapted to the calculation of the realized subniche S_*R*_. E_*1*_ and E_*2*_ are the environmental gradients calculated after an ordination technique. **E** is the realized environmental space (filled light blue minimum convex polygon). **N**_*R*_ is the species realized niche (dotted orange contour). **K** is the subset realized environmental space (dark blue minimum convex polygon). **S**_*P*_ is the existing fundamental subniche (the yellow contour)—a union of **S**_*B*_ and **S**_*R*_. **S**_*B*_ is the subset biotic reducing factor (the part of **K** found within the orange contour), or biological constraint, and **S**_*R*_ is the realized subniche (the green minimum convex polygon).

However, the lack of study on the role of biotic interactions (e.g., competition, predation, mutualism, dispersal, and colonization) is a major limitation for defining species’ niches appropriately (Davis et al., 1998; Soberón & Nakamura, 2009). Studies have shown that incorporating biotic factors can lead to better predictions of species’ distributions (Heikkinen et al., 2007), yet, despite this evidence, biotic factors are still underused and greater assessment is required to fully understand species’ niche dynamics (Soberón & Nakamura, 2009). According to Jackson & Overpeck (2000), the constraints exerted on the realized niche by biotic process are the differences between the potential niche (i.e., the intersection between the fundamental niche and the realized environmental space (Soberón & Nakamura, 2009)) and the realized niche; the realized environmental space being “the portion of the total *n*-dimensional environmental space that is actually represented […] within a specified region at a given time” (Jackson & Overpeck, 2000). Later on, the potential niche was renamed “the existing fundamental niche” by Peterson et al. (2011). Therefore, the biotic interactions are the differences between the existing fundamental niche and the realized niche. The role of biotic interactions is not directly measurable by observation, as it requires an estimation of the fundamental niche. However, in order to estimate biological interactions, adaptation of the concept of the existing fundamental niche concept can be applied to the decomposed realized niche, which can be measured by observation. This concept requires the decomposition of the realized environmental space, **E**, into subsets of the realized environmental space, **K**, so that **K** is a subset of **E** (Fig. 1). **K** represents the available conditions found within **E**, at a smaller time and/or spatial scale than in **E**. Now considering **N**_*R*_, the realized niche, found within **E**, as the best estimation of the “fundamental niche” of the species under **K**, the intersection between **K** and **N**_*R*_ represents the existing fundamental subniche, **S**_*P*_ (Fig. 1). The existing fundamental subniche corresponds to the abiotically reduced part of **N**_*R*_ by **K**. Therefore, **S**_*P*_ includes the subset biotic factor, **S**_*B*_, reducing **S**_*P*_ into the realized subniche, **S**_*R*_ (Fig. 1). In summary:

$${{\bf{S}}_R}\bigcup \,{{\bf{S}}_B}{\bf{\,= \, }}{{\bf{S}}_P}{\bf{ \, = \, }}\;{\rm{K }}\bigcap \,{{\bf{N}}_R}$$

**S**_*B*_ can be caused by negative biological interactions (e.g., predation, competition, parasitism, etc.) but also can be due to dispersal limitation from the species itself (i.e., lack of time for migration) or occupancy by another species (Peterson et al., 2011) (Fig. 1).

The realized niche can be measured directly from the *n*-dimensional hypervolume (Blonder et al., 2014) but ordination techniques are also well suited to investigate species and environmental relationships. The outlying mean index (OMI) analysis is an ordination technique designed to explicitly take into account the ecological niche of each species within a community (Dolédec, Chessel & Gimaret-Carpentier, 2000). The OMI analysis seeks combinations of environmental variables that maximize average species marginality, i.e., the squared Euclidean distance between the mean habitat conditions used by a species and the mean habitat conditions of the sampling domain (the sampling domain can be defined on a temporal and/or spatial scale). Ecologically, as Hernández-Fariñas et al. (2015) stipulated “species with high values have marginal niches (occur in less common habitats in the studied region), and those with low values have non-marginal niches (occur in typical habitats in the region).” In other terms, in OMI analysis, the position of each species in the multidimensional space depends on its niche deviation from a uniformly distributed theoretical species, which would occur under all available habitat conditions (i.e., ubiquitous). In addition, the technique provides information on species’ niche breadth or tolerance where “high tolerance values are associated with taxa occurring in a wide range of environmental conditions (generalist taxa) while low values of tolerance imply that the taxa are distributed across a limited range of environmental conditions (specialist taxa)” (Hernández-Fariñas et al., 2015).

**Figure 2:**
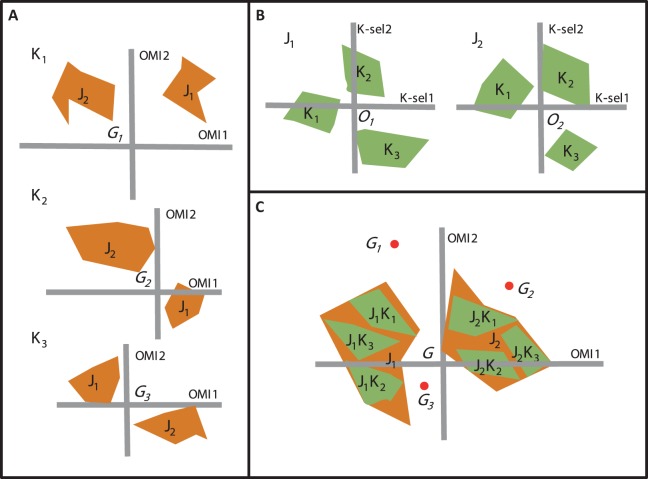
The difference between the OMI analysis, K-select analysis and WitOMI calculations. (A) OMI analyses performed on three hypothetical subsets (*K_1_*, *K_2_*, and *K_3_*) and two species (*j_1_*, *j_2_*). The three positions of the two species niches with their corresponding minimum convex polygon (i.e., niche breadth) are not comparable across subsets (*K_1_*, *K_2_*, and *K_3_*) because ordination is performed for each subset, creating new origins, *G_1_*, *G_2_*, and *G_3_* (i.e., equivalent to the average habitat conditions used by the community). (B) Separate *K*-select analyses performed for each species, *j_1_* and *j_2_*, in the three subsets, *K_1_*, *K_2_*, and *K_3_*. The resulting niches for each subset of the two species are not comparable because the origins *O_1_* and *O_2_* represent the average habitat used by the species *j_1_* and *j_2_*, respectively. (C) Species’ niche position and breadth analyzed with the OMI analysis. WitOMI, further decompose the species niche into subniches (*j_1_ K_1_*, *j_1_ K_2_*, *j_1_ K_3_* and *j_2_ K_1_*, *j_2_ K_2_*, *j_2_ K_3_* for *j_1_* and *j_2_*, respectively) and indexes can be calculated from *G*, WitOMI*G*. The black dots (*G_1_*, *G_2_*, and *G_3_*), representing the average subset used by one assemblage, are used to calculate subniche indexes, WitOMI*G_K_*.

Beside OMI analysis, the *K*-select analysis is another ordination technique which is also based on marginality (Calenge, Dufour & Maillard, 2005). The *K*-select analysis consists of a non-centered principal component analysis calculated on a table containing the marginality vector coordinates of a species population for the habitat variables (Calenge, Dufour & Maillard, 2005). The output of the *K*-select analysis is a multicollinearity of habitat variables for which the marginality is the greatest; in other words, a synthesis of the variables which contribute most to habitat selection. The main difference between the two techniques concerns the weighting of the sampling units (SUs), i.e., one unit of the sampling domain. The OMI analysis assumes the equal availability of SUs (i.e., colonizable) to all monitored species regardless of time and/or space, whereas the *K*-select analysis considers an equal availability of SUs within each subset (i.e., group of SUs) of the sampling domain (e.g., seasons within a year or sites within a region for one species) (Thomas & Taylor, 1990). Let us consider an assemblage of two species (*j_1_* and *j_2_*) collected within a sampling domain divided into three subsets (*K_1_*, *K_2_*, and *K_3_*). To study species’ niche dynamics within the community over the three subsets, one can perform three separate OMI analyses, i.e., one for each subset (Fig. 2A) or two *K*-select analyses, i.e., one for each species (Fig. 2B). However, whichever of the two analyses used, a new environmental gradient is created for each analysis performed.

To study niche dynamics, some researchers have used several distinct OMI analyses on habitat condition subsets. For example, Grüner et al. (2011) performed 40 OMI analyses (one per year) on a time series of three phytoplankton species to depict their temporal niche trajectories. Hof, Rahbek & Araújo (2010) performed 14 OMI analyses (one per region and per family) to assess the phylogenetic relatedness between different amphibian families and genera within each geographical region. Mérigoux & Dolédec (2004) performed two OMI analyses on freshwater invertebrates (one per season, spring and autumn) to address seasonal shifts in the hydraulic niche of taxa. One drawback of these approaches lies in the available habitat conditions (as defined by Dolédec, Chessel & Gimaret-Carpentier (2000)), which may greatly vary between each subset, impacting the calculations of indexes such as marginalities and tolerances (Fig. 2A). As a result, the observed changes in marginalities can be partly attributed to temporal (annual for Grüner et al. (2011) and seasonal for Mérigoux & Dolédec (2004)) or spatial changes (Hof, Rahbek & Araújo, 2010) in average habitat conditions used by taxa in the assemblage. Hence, performing separate OMI analyses on different habitat condition subsets, using the same domain of habitat conditions, does not make the species’ niches comparable across subsets, because average habitat conditions most likely vary from one subset to another. To our best knowledge, *K*-select analyses have not yet been performed on species assemblages, but rather on several populations of one species (reindeer) (Pape & Löffler, 2015). In this case study, the authors performed nine *K*-select analyses (one population per season), creating nine different habitat gradients (Pape & Löffler, 2015). However, the average habitat conditions used changed for each ordination, giving different meanings to the marginality values for each analysis, making comparisons between seasons inaccurate.

Here, our main goal is to provide a method to estimate the dynamics of the realized subniches, **S**_*R*_, of each species of an assemblage, compared to *G*, representing the overall average habitat condition found in **E**. Furthermore, the subniche can also be compared to *G_K_*, which represents the average subset habitat conditions found in **K**. We therefore, propose to combine the properties of the OMI analysis (maximizing the average species marginality within a community) and the *K*-select marginality decomposition within a species (maximizing the species marginality within subsets, i.e., the subniche). Our proposal allows comparing the ecological niche and ecological subniches of species in the *n*-dimensional environmental space, by fixing the ecological conditions using the OMI analysis (Fig. 2C) and then decomposing the occupation of the realized niche in the same manner as the preliminary calculations of *K*-select analysis. In addition, it describes the possible subniche shift and/or conservatism of species within an assemblage across temporal and/or spatial subsets within the habitat conditions of the sampling domain. Finally, the difference between the existing fundamental subniche, **S**_*P*_, and the realized subniche, **S**_*R*_, would therefore correspond to the observed biological constraint, **S**_*B*_. We illustrate the potential of this method using published studies including both a temporal case (seasonality; see Mérigoux & Dolédec (2004)) and a spatial case (longitudinal stream gradient; see Dolédec, Chessel & Gimaret-Carpentier (2000)).

## The Witomi Concept

The OMI measures the marginality of a species (i.e., the weighted average of SUs used by the species) from the average condition of the sampling domain, *G* (Dolédec, Chessel & Gimaret-Carpentier, 2000). OMI originates from the combinations of **Z**_0_, the standardized environmental variable table, and **Fr**, the species frequency table. Here, we aim to estimate the niche occupation dynamics of each species within the community, at different subsets of habitat conditions within the sampling domain. In other words, we aim to scrutinize the subniches of species within a community in the same reference plane, made by the resulting factorial axes from the OMI analysis. The subniche is defined hereafter, as a subset of habitat conditions used by a species.

Inspired by the OMI analysis (Dolédec, Chessel & Gimaret-Carpentier, 2000) and the decomposition of marginalities used in *K*-select analysis (Calenge, Dufour & Maillard, 2005), we propose to calculate two additional marginalities. First, the WitOMI to *G* (WitOMI*G*) is the species marginality (i.e., the weighted average of SUs of a given subset used by the species) to the average habitat conditions of the sampling domain (*G*; see Eq. S9 in Appendix S1). Second, the WitOMI to *G_K_* (WitOMI*G_K_*) is the species marginality compared to the average habitat condition used by the community in a *K* subset habitat conditions (*G_K_*; see Eq. S20 in Appendix S1).

To obtain WitOMI*G*, we first calculate the species frequency relative to each **K** subset (with 1 ≤ **K** ≤ *N*). Second, the *N* **Fr**_*K*_ matrices are concatenated to yield the overall species frequency table (Fr*). Third, the standardized environmental table **Z**_0_ is used in combination with (Fr*) to calculate WitOMI*G* following the Eq. (S9) in Appendix S1.

The calculation of WitOMI*G_K_* first requires centering each of the *K* subsets of the standardized environmental table **Z**_0_(*n* × *p*), independently yielding several matrices ${\bf{Z}}_{K^*}$. The *N* ${\bf{Z}}_{K^*}$ are then concatenated to yield another environmental table Z*. Finally, Z* is used in combination with (Fr*) to calculate WitOMI*G_K_* following the Eq. (S20) in Appendix S1.

Outlying mean index analysis is then used as the reference ordination technique. The subniche coordinates in the *n*-dimensional space, ℝ^*p*^, are projected onto the OMI factorial plane by multiplying their values by the corresponding eigenvectors. As a result, the niche and the subniche parameters (marginality and tolerance) of the species are all in the same reference factorial plane.

Within outlying mean index to *G* and the WitOMI*G_K_* calculations are shown in Appendix S1, and do not include the OMI calculations and the OMI analysis, which are fully described in Dolédec, Chessel & Gimaret-Carpentier (2000). The WitOMI calculations, as well as other computational tools, are available in the “subniche” package for R software (R Core Team, 2013) and can be downloaded for free at the http://cran.r-project.org. The “subniche” tutorial is available at https://github.com/KarasiewiczStephane/WitOMI.

### Statistical significance

The statistical test for significance of the species marginality in the *K* subsets, which is inspired from Dolédec, Chessel & Gimaret-Carpentier (2000), uses a Monte Carlo test (Manly, 1991). First, the significance of the subset habitat conditions *K* was calculated by considering the equiprobability of *n*! permutations of the habitat conditions table *Z*_0_. We compared the observed average of subset habitat conditions, *G_K_*, to the distribution of the 1,000 permutations values following the null hypothesis that *G_K_* is not different from overall average habitat conditions, represented by *G*.

The significance of the species marginalities from the average habitat condition *G*, WitOMI*G*, and from the average subset habitat conditions *G_K_*, WitOMI*G_K_*, were calculated by considering the equiprobability of *k*! permutations of the species profile **Fr**_*K*_. Second, a comparison of the observed WitOMI*G* (Eq. S9 in Appendix S1), and WitOMI*G_K_* (Eq. S20 in Appendix S1) with the distribution of the 1,000 permutations values, found under the *K* subset, following the null hypothesis that the species within a subset is uninfluenced by its overall average habitat conditions (ubiquitous), for WitOMI*G* and by subset habitat conditions for WitOMI*G_K_*, respectively. Third, the means of the observed WitOMI*G* and WitOMI*G_K_* across the *K* subset were compared to their respective simulated mean.

### Graphical display

**Figure 3:**
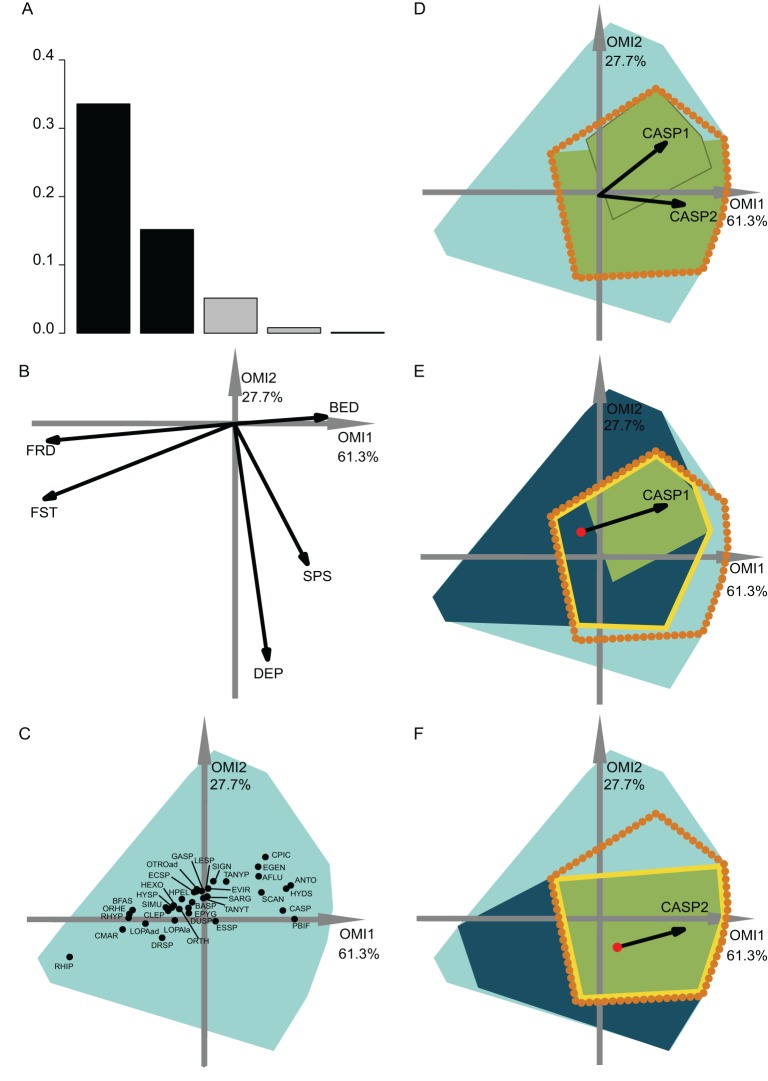
OMI analysis of the invertebrate community and the WitOMI. (A) Bar chart of the eigenvalues, measuring the mean marginality explained by each factorial axes. The black bars are the chosen factorial axis, OMI1 and OMI2. (B) Canonical weights of environmental variables (FRD, froude; FST, hemisphere number; BED, bed roughness; SPS, substratum particle size, DEP, depth). (C) Representation of the statistically significant species’ realized niche positions on the first two factorial axes (Appendix S2; Table S1) (see codes in Appendix S2; Table S3). The light blue minimum convex polygons represent the habitat conditions constraint of all SUs domain. (D) The realized subniches dynamism of *Caenis* sp. (CASP) is the green minimum convex polygon, subsetting the realized niche, the orange dotted polygon. The arrows represent the WitOMI*G*. (E and F) Represent the *Caenis* sp. Realized subniches under the subset habitat conditions *K*, the dark blue polygon, subsetting the existing fundamental subniche (the yellow contour), encountered in spring and autumn for (E) and (F), respectively. The red dots represent the suborigin, *G_K_* and the arrows represent the WitOMI*G_K_*.

**Figure 4:**
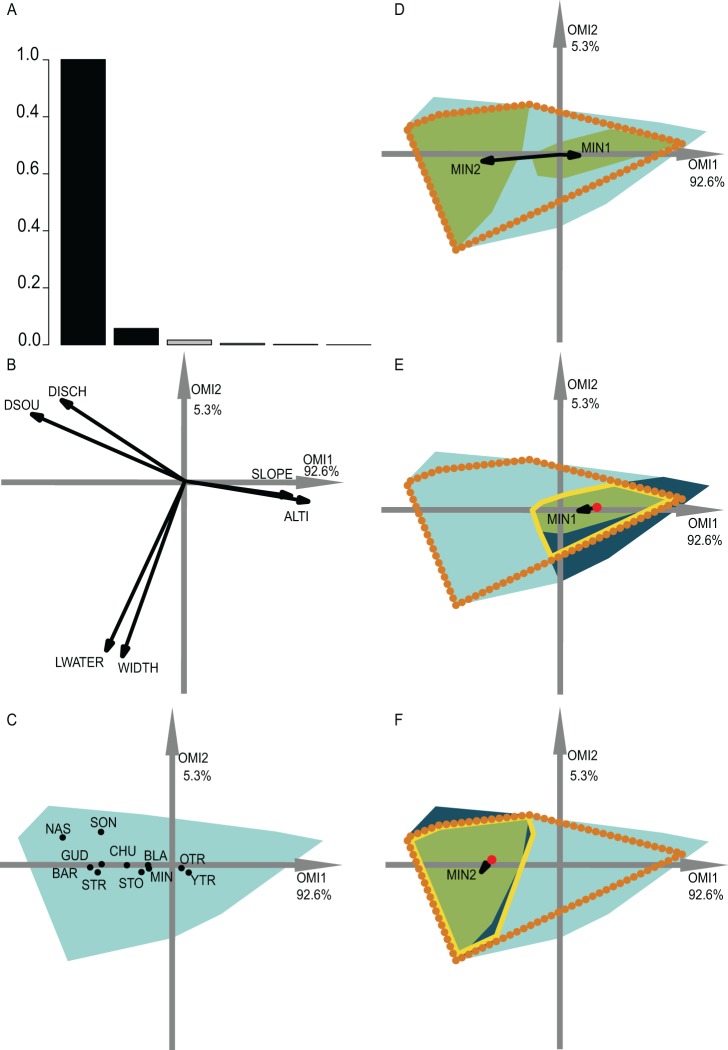
OMI analysis of the streamfish and the WitOMI. (A) Bar chart of the eigenvalues, measuring the mean marginality explained by each factorial axes. The black bars are the chosen factorial axis OMI1 and OMI2. (B) Canonical weights of environmental variables (DSOU, distance to the source; DISCH, mean annual discharge; LWATER, lowest monthly discharge occurring every five years; WIDTH, mean stream width; SLOPE, slope; ALTI, altitude). (C) The realized niche position on the first two factorial axes of the significant species (Appendix S2; Table S2) (see codes in Appendix S2; Table S4). The light blue minimum convex polygons represent the habitat conditions constraint of all SUs domain. (D) The realized subniche dynamics of Minnow (*Phoxinus phoxinus*) are the green minimum convex polygons, subsetting the realized niche, the orange dotted minimum convex polygon. The arrows represent the WitOMI*G*. (E and F) Represent the Minnow (*Phoxinus phoxinus*) realized subniches under the habitat conditions constraint, the dark blue minimum convex polygon, subsetting the existing fundamental subniche (the yellow contour), encountered upstream and downstream for (E) and (F), respectively. The red dots represent the suborigin *G_K_* and the arrows the WitOMI*G_K_*.

The graphical display of the species’ realized niche and subniche can be obtained by projecting the available SUs of matrix **Z**_0_ on the first two factorial axes of the OMI analysis (OMI1 and OMI2 in Figs. 3 and 4),

$${\bf{Z}}_0^{\bf{u}} = \bf{Z}_0 \times {\bf{u}}$$

with **u** being the eigenvectors chosen after the OMI analysis and **Z**_0_^**u**^ corresponding to the matrix of coordinates of all available SUs projected onto the OMI analysis plane.

The graph origin is the center of gravity of all available SUs, *G*, which represents mean overall habitat conditions. Similarly, the subset origin, *G_K_*, is the barycenter of available *k* SUs within the *K* subset, since ${\bf{Z}}_{K^*}$ is centered. The species niche and subniche positions correspond to the weighted mean of coordinates, whose weight is equal to the species frequency (see section “species frequency table,” Appendix S1). Finally, the minimum convex polygon’s contour of available SUs (black in Figs. 3 and 4) and of used SUs (blue and the purple dotted and dashed in Figs. 3 and 4) complete the realized niche and subniche breadth representation of species. The minimum convex polygons were drawn with the package “ade4” for R software (Dray & Dufour, 2007). The species niche and subniche positions and their respective minimum convex polygons, relative to the origins, give us an idea about the habitat conditions used by species within the constraining habitat highlighted by the OMI analysis.

## Ecological Application

To illustrate the potential of combining the OMI analysis with the WitOMI we used two data sets that address the question of subniche dynamics according to temporal or spatial characteristics of the habitat.

### Temporal subniche dynamics

The first data set investigated the hydraulic requirement of 57 invertebrate taxa (Mérigoux & Dolédec, 2004). Herein, instead of performing an OMI analysis for each season (i.e., spring and autumn as done by authors) we performed one for the entire year. A total of 35 out of 57 taxa had significant OMI (Appendix S2; Table S1). We selected the first two OMI axes, which represented 89% of the explained variability (Fig. 3A), in order to represent the subniches. As depicted by Mérigoux & Dolédec (2004), the first axis shows that FST hemisphere number and Froude number are the most influential hydraulic parameters on the species’ realized niche (Figs. 3B and 4C). WitOMI were then calculated for spring and autumn for each of the 35 significant species. All WitOMI (WitOMI*G* and WitOMI*G_K_*) were significant (Appendix S2; Table S1).

As an example, *Caenis* sp. used an uncommon habitat (OMI = 2.09) compared to the rest of the community (Appendix S2; Table S1). *Caenis* sp. has a preference for high bed roughness compared to most species (Figs. 3B and 3C). A similar pattern can be found with its realized subniches (WitOMI*G* = 2.28 and 2.24 for spring and autumn, respectively) (Appendix S2; Table S1). The realized subniche positions demonstrate a shift, seemingly caused by the increasing depth in autumn (Fig. 3D). *Caenis* sp. tolerance also showed an increase from spring to autumn (Tol = 0.45 and 0.75, respectively) (Appendix S2; Table S1). Now considering each season separately, *Caenis* sp. occupied different parts of its realized niche (Figs. 3E and 3F). *Caenis* sp. thus used a more atypical habitat compared to the one used by the assemblage in spring and autumn (WitOMI*G_K_* = 2.44 and 2.46) (Appendix S2; Table S1). Despite the seasonal habitat change, the marginality of the habitat used by the species stayed similar. The tolerance also increased when considering the two habitat conditions separately (Tol = 0.46 and 0.75). *Caenis* sp. occupied a greater part of its existing fundamental subniche in autumn than in spring, which suggests more appropriate abiotic conditions or less constraint by biotic interactions.

In spring, the *Caenis* sp. realized subniche (the green minimum convex polygon, Fig. 3E) did not fully occupy the intersection between the niche (orange dotted contour) and the subset habitat condition (dark blue minimum convex polygon) (i.e., existing fundamental subniche). Herein, the empty part of the existing fundamental subniche therefore corresponds to the biological constraint exerted on the species realized subniche. The decreasing biological constraint exerted on the *Caenis* sp. realized subniche from spring to autumn seems to be correlated with the decreasing number of species having a significant marginality (35 to 23 from spring to autumn).

### Spatial subniche dynamics

The second data set investigated the fish assemblages used by Dolédec, Chessel & Gimaret-Carpentier (2000). We selected the first two OMI axes, which represented 97.9% of the explained variability, in order to represent the realized subniches (Fig. 4A). We divided the data along the first axis, which is mostly defined by altitude and slope, considering distinct upstream and downstream habitat conditions. All of the WitOMI (WitOMI*G* and WitOMI*G_K_*) were significant (Appendix S2; Table S2).

As an example, minnow (*Phoxinus phoxinus*), was distributed over the entire longitudinal gradient and used common habitat (OMI = 0.45). However, the used habitat was more marginal downstream than upstream (WitOMI*G* = 0.33 and 4.61 for upstream and downstream, respectively) (Fig. 4D) (Appendix S2; Table S2). In addition, we observed a shift in the species’ marginality and tolerance within its realized niche (Fig. 4D). The reason for the realized subniche change in marginality and tolerance can be explained by the difference between upstream and downstream subset average habitat conditions (red dot, Figs. 4E and 4F) and subset habitat condition constraints (dark blue minimum convex polygon, Figs. 4E and 4F), impacting the species’ realized niche. Focusing on the upstream and downstream habitat conditions separately, minnow’s marginality upstream was higher than downstream (WitOMI*G_K_* = 0.3 and 0.04 for upstream and downstream, respectively) (Appendix S2; Table S2). In both conditions, the species used a similar habitat to the one used by the assemblage. Furthermore, upstream conditions seemed to have greater constraint on the species realized niche occupation, contracting the minnow realized subniche breadth (Fig. 4E), whereas downstream conditions allowed the species to occupy a greater part of its existing fundamental subniche (Fig. 4F).

In addition, both young and adult trout were found along the entire longitudinal gradient with a preference for upstream conditions (WitOMI*G_K_* = 0 for old and young trout, respectively) (Appendix S2; Table S2). Minnow, stone loach and chub were mostly found downstream while the nase, southwestern nase and streambleak species were exclusive to downstream average habitat conditions (Appendix S2; Table S2). These results were coherent with those of Dolédec, Chessel & Gimaret-Carpentier (2000) on the same data set (Fig. 4C). In addition, WitOMI showed that the conditions found downstream offered greater habitat variability because other environmental variables, aside from altitude and slope, influenced species’ subniches. The greater variability of habitat downstream permitted hosting more species than upstream, where trout appeared to use most of the habitat conditions.

## Discussion

The WitOMI offer new interpretations to niche dynamics by considering subsets of habitat conditions within which the species’ realized subniches are developed. WitOMI complement the OMI approach by shifting how realized niches are perceived along fluctuating habitat conditions. WitOMI make all realized subniches comparable along the same environmental gradient as they all refer to the same OMI analysis. The realized subniche parameters can be explained by the average habitat conditions used by the assemblage over the entire sampling domain, WitOMI*G*, and by the average habitat conditions used within a subset of SUs WitOMI*G_K_*. The advantage of decomposing the realized niche into realized subniches is that the WitOMI are simple measures, which integrate the species realized subniche specialization from the habitat studied (WitOMI*G*) and from the decomposed habitat (WitOMI*G_K_*), giving additional hints on the role played by different environmental variables. However, our approach has the same experimental limitation as the OMI analysis. The environmental variables used may not be sufficient to define the realized niche parameters, making the decomposition of the realized niche into realized subniches irrelevant.

**Figure 5:**
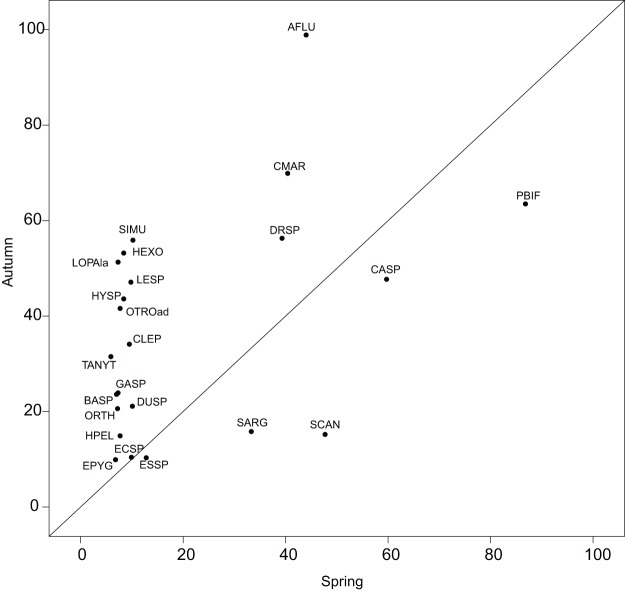
Within outlying mean index to *G* values (as percentage of the total variability, Appendix S2; Table S1) of the 23 significant taxa common to both seasons. Names are abbreviated using codes as given in Appendix S2; Table S3.

The reference species, which represents a theoretical ubiquitous species using the overall average habitat conditions of a sampling domain, helped quantify the shift in realized subniches. The utilization of *G* as a reference smoothens the atypical conditions, avoiding an over-interpretation of habitat condition effects on the species’ niches. Reconsidering the results of Mérigoux & Dolédec (2004), who performed a separate OMI analysis on each season, we found less species common to both seasons with a significant marginality (23 herein and 35 in Mérigoux & Dolédec (2004)). Nonetheless, the pattern found in Fig. 5 was similar to the one found in Fig. 2 of the authors, i.e., with more species in autumn having significant marginality than in spring, thus underlying the fluctuating effect of hydraulic constraints advocated by the authors (Appendix S2; Table S1). The WitOMI thus provide more relevant comparable values. In addition, the use of *G_K_*, which can be representative of more variable conditions, can provide additional information about the environmental variables driving the species niche and community composition.

However, the method is limited by the number of SUs defining the sampling domain. This limitation underlines the inapplicability of the WitOMI to an unsignificant realized niche of the OMI analysis. WitOMI are also limited by the number of subsets used to decompose the sampling domain. In the ecological application, we used two subsets of habitat conditions to decompose the realized niche into two realized subniches. The *K* SUs defining a subset have an impact on the subniche parameters’ significance. Even if it was not the case in our study, a low number of SUs within subsets can cause the test of significance to give a low probability of estimating subset habitat conditions (*G_K_*).

**Figure 6:**
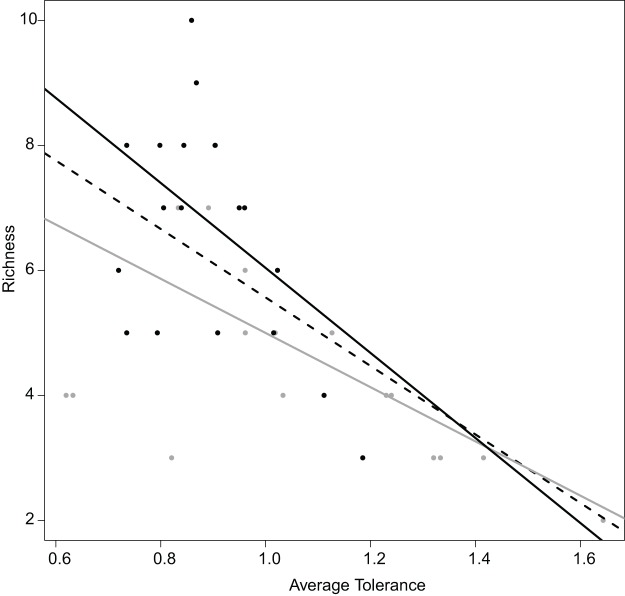
Relation between the average tolerance values of sites upstream and downstream, and their fish species richness. Overall, dashed black line, *R*^2^ = 0.64 with *P* < 0.001; upstream, grey line, *R*^2^ = 0.68 with *P* < 0.001; downstream, plain black line, *R*^2^ = 0.21 with *P* = 0.034.

Realized subniches can be compared to their respective subset origins, the subset theoretical ubiquitous species using the most general subset of habitat conditions, in how they differ from *G*. This comparison provides a more detailed interpretation in the realized niche shift. For instance, similar to Dolédec, Chessel & Gimaret-Carpentier (2000), there was a negative relationship between species richness and realized niche breadth (Fig. 6). The negative relationship was greater upstream (*R*^2^ = 0.68 and 0.21 for upstream and downstream, respectively) (Fig. 6). In other words, there was increasing competition upstream because the most common species (with the lowest WitOMI*G_K_*) found upstream, the trout (WitOMI*G_K_* = 0 for upstream), has a broad realized subniche upstream (Tol = 1.62 and 1.09 for upstream and downstream, respectively), which decreases species diversity (8 and 11 species for up and downstream, respectively). In this spatial example, the WitOMI*G_K_* allows assessing which species were common upstream, giving a more accurate description of the fish distribution pattern (Dolédec, Chessel & Gimaret-Carpentier, 2000), and community structure.

The subsets of habitat conditions and the two WitOMI can be tested with random permutations to assess whether subset habitat conditions and the species marginality are significantly different from what would be expected by chance. They follow the null hypothesis that the subset habitat conditions *G_K_* are not different from the overall habitat conditions *G*, and that a species is not influenced by habitat conditions for WitOMI*G*, or by the subset of habitat conditions for WitOMI*G_K_*. Lack of significance in the permutation test can be explained by the defined subset conditions, which might not be appropriate enough, making *G_K_* weakly relevant and the WitOMI unsignificant. This emphasizes the need for a reference habitat condition and a significant realized niche **N**_*R*_ (e.g., OMI analysis), which can be further decomposed to study realized subniche dynamics. The total inertia of the species (see Eq. 13 in Appendix S1) characterizes the decomposition of the realized niche, **N**_*R*_, calculated with the OMI analysis, into the species realized subniches, **S**_*R*_, within the subset habitat conditions, *G_K_*.

The decomposition of the realized niche allows estimating the biological constraints, **S**_*B*_, exerted on a species (e.g., *Caenis* sp.) in our temporal example. The comparison between the subniche, **S**_*R*_, and the existing fundamental subniche, **S**_*P*_, revealed an unused part of **S**_*P*_ which can be attributed to biological constraints. The quantification of biological constraints is dependent on the envelope chosen to represent the niches and subniches. Quoting Guisan et al. (2014), the niche envelope is “the envelope of conditions in multivariate environmental space defining a species niche. The boundary of the envelope can be defined in many different ways, e.g., percentiles; Broennimann et al. (2012).” In this study we used the minimum convex polygon. Therefore, our quantification of the biological constraints, **S**_*B*_, consisted of measuring the difference between the area of **S**_*P*_ and **S**_*R*_. The biological constraints can be given in percentage of the **S**_*P*_ area but is the minimum convex polygon truly the best envelope? For example, Blonder et al. (2014) developed a method to calculate the *n*-dimensional hypervolume which can be used to quantify the hypervolume of **N**_*R*_, **S**_*R*_, **S**_*P*_, and the biological constraints. As suggested by Blonder et al. (2014), hypervolume might have holes, which may be the equivalent of the biological constraints of a species niche estimated, within the *n*-dimensional hypervolume. This perspective could bring further insight into the invasive species strategy as explained in Blonder (2016). **S**_*B*_, which is now quantifiable under subset habitat conditions, can be of a different nature. It can either be due to negative biological interactions, or dispersal limitation (Peterson et al., 2011). As a result, caution should be taken while interpreting the nature of **S**_*B*_.

The description of the subset conditions of the different variables can reveal how the community responds to changing habitat conditions. We can imagine the case where the shifted species’ realized subniches do not shift in the same direction as the suborigins. What mechanisms would be involved in species realizing their niches? Would the community be threatened by a changing environment? These questions emphasize the need for using the WitOMI that enables comparing different species’ realized niches in a community under changing habitat conditions. Our proposed refinement of the OMI analysis allows us to make hypotheses on the mechanisms involved in a species realizing its niche. The ecophysiological requirements of species should vary with changing habitat conditions, since species must respond to the environmental variation in order to survive. Kleyer et al. (2012) recently developed this idea using the outlying mean index followed by generalized additive modeling (OMI-GAM). First, the method consists of using the OMI analysis to determine the species’ responses to habitat conditions and their realized niche positions and breadths. Second, traits are used as explanatory variables in a GAM to explain the above species responses. The OMI-GAM thus answers the question “How do trait expressions of species respond to environmental gradients?” Similarly, the WitOMI could be used as a first step of OMI-GAM to study trait expressions within different habitat conditions and to reveal shifts in species life-strategies via a change in the functional trait hierarchy.

The main strength of WitOMI is that they can be applied to any species, population, community, or ecosystem. Regarding the previous example, reanalyzing the data with the WitOMI, should improve the accuracy and details of the results (Hof, Rahbek & Araújo, 2010; Grüner et al., 2011; Pape & Löffler, 2015). This proposal can be used in various aspects of ecology, such as the structure and dynamics of populations and interactions among individuals of the same or different species. In the context of global change, the methods can reveal the response of individuals and groups of organisms, and the organization of biological communities (Hof, Rahbek & Araújo, 2010; Grüner et al., 2011). The WitOMI can be used as a statistical basis for future ecological niche models such as modeling the potential of an invasive species to establish itself in a new ecosystem (Broennimann et al., 2012; Guisan et al., 2014). As a perspective, the WitOMI can be applied to study community responses to environmental change, including the impacts of possible community resource-competition.

## Supplemental Information

10.7717/peerj.3364/supp-1 Supplemental Information 1 Within Outlying Mean Indexes Calculations. Mathematical description of the WitOMI parameters.

10.7717/peerj.3364/supp-2 Supplemental Information 2 WitOMI results and specie’s code. Tables S1 and S2 are the results of the OMI analysis, the value of the WitOMI parameters and their respective *P* values, for species of both ecological examples. Tables S3 and S4 are the respective code for each species of both ecological examples.

## Additional Information and Declarations

### Data Availability

The following information was supplied regarding data availability: The data used are RDA files and are named “ardecheinv” and “drome.” The data, along with the WitOMI calculations as well as other computational tools, are available in the “subniche” package and can be downloaded for free on the CRAN repository http://cran.r-project.org or on Github (https://github.com/KarasiewiczStephane/WitOMI).
