# Supplementary material for: Within outlying mean indexes: refining the OMI analysis for the realized niche decomposition
Source: PeerJ. 2017 May 23;5:e3364. doi: 10.7717/peerj.3364 (PMC5444369; doi:10.7717/peerj.3364)
Supplement: Supplemental Information 1 — Mathematical description of the WitOMI parameters. [file peerj-05-3364-s001.pdf]

APPENDIX S1: WITOMI CALCULATIONS

Species frequency table

Here and elsewhere, we follow the mathematical notations used by Dolédec et al. (2000). Let us extract $\mathbf{Y}_K(k\ samples \times t\ species)$, from the faunistic table $\mathbf{Y}(n\ samples \times t\ species)$ with $1 \leq k \leq n$. Let us transform subset $\mathbf{Y}_K$ into a species profile table (noted $\mathbf{Fr}_K$) that contains the frequency of species for each SUs, $f_{K_{i_s/j}}$ as follows:

$$f_{K_{i_s/j}} = \frac{y_{K_{i_s j}}}{y_{K_{.j}}} \qquad 1 \leq i_s \leq k,\ 1 \leq j \leq t \tag{S.1}$$

where $y_{K_{i_s j}}$ is the abundance of species j in SU i_s and $y_{K_{.j}}$ the column total of species j equal to

$$y_{K_{.j}} = \sum_{i_s=1}^{k} y_{K_{i_s j}} \tag{S.2}$$

Then the species profile table $\mathbf{Fr}*$ concatenates $\mathbf{Fr}_K$ as follows:

$$\mathbf{Fr}* = \begin{bmatrix} \mathbf{Fr}_1 \\ \vdots \\ \mathbf{Fr}_K \\ \vdots \\ \mathbf{Fr}_N \end{bmatrix} \quad 1 \leq K \leq N \tag{S.3}$$

with N the number of subsets.

Subniche parameters calculated from the origin G

The center of gravity (G) of SUs is at the origin of the axes of the OMI analysis and corresponds to the overall mean habitat conditions used by the taxa in the assemblage (Dolédec et al., 2000). Let us consider N subsets habitat conditions of the environmental table $\mathbf{Z}_0$ equals to:

$$\mathbf{Z}_0 = \begin{bmatrix} \mathbf{Z}_1 \\ \vdots \\ \mathbf{Z}_K \\ \vdots \\ \mathbf{Z}_N \end{bmatrix} \quad 1 \leq K \leq N \tag{S.4}$$

Let us extract $\mathbf{Z}_K(k \times p)$, a matrix of $\mathbf{Z}_0(n \times p)$, having k rows, with $1 \leq i_s \leq k$ and p variables (Figure 3).

Let the faunistic frequency table, $\mathbf{Fr}_K(k \times t)$ contains the frequency of t species in the k SUs. M_i represents SU i of table $\mathbf{Z}_0$ in the multidimensional space $\mathbb{R}^p$. Let consider $M_{K_{i_s}}$, representing SU i_s of table $\mathbf{Z}_K$ in the same multidimensional space $\mathbb{R}^p$. The total inertia of table $\mathbf{Z}_K$ equals:

$$I_{T_K} = \sum_{i_s=1}^{k} p_{K_{i_s}} \parallel M_{K_{i_s}} \parallel^2_{\mathbf{I}_p} \tag{S.5}$$

with $p_{K_{i_s}}$ being the weight of SU i_s. The inertia of species j considering the matrix $\mathbf{Z}_K$ equals:

$$I_{T_K}(j) = \sum_{i_s=1}^{k} f_{K_{i_s/j}} \parallel M_{K_{i_s}} \parallel^2_{\mathbf{I}_p} \tag{S.6}$$

The inertia $I_{T_K}(j)$ represents the total inertia of $\mathbf{Z}_K$ weighted by the species j profile. Similarly to the proposal of Dolédec et al. (2000), the SUs i_s that do not have species j do not add to the species j inertia. Let consider a $\mathbf{I}_p$-normed vector $\mathbf{u}_K$ ($\| \mathbf{u}_K \|^2_{\mathbf{I}_p} = 1$). The projection of the k rows of the matrix $\mathbf{Z}_K$ onto the vector $\mathbf{u}_K$ results in a vector of coordinates $\mathbf{Z}_K\mathbf{u}_K$. Therefore, the average position of species j on $\mathbf{u}_K$, equivalent to the center of gravity of species j, is defined as:

$$T_{K_j} = \mathbf{f}_K^\top \mathbf{Z}_K \mathbf{u}_K \qquad \mathbf{f}_K^\top = (f_{K_{1/j}}, \ldots, f_{K_{i_s/j}}, \ldots, f_{K_{k/j}}). \tag{S.7}$$

With Eq. S8, marginality within a subset of habitat conditions, or within-subset outlying mean index (WitOMIG) of species j [noted $m_{a_K}(j)$] along $\mathbf{u}_K$ equals:

$$m_{a_K}(j) = T_{K_j}^2 = (\mathbf{f}_K \mid \mathbf{Z}_K\mathbf{u}_K)^2_{\mathbf{I}_p} = (\mathbf{Z}_K^\top \mathbf{u}_K \mid \mathbf{f})^2_{\mathbf{I}_p} \tag{S.8}$$

This marginality represents the deviation between the average position of species j within subset K from the origin (G). Also equivalent to the distance between the subset average habitat conditions used by species (j) and the overall average habitat conditions found in the area (G).

From Eq. S9, the maximization of $m_{a_K}(j)$ has for solution $\mathbf{u}_K$ equal to:

$$\mathbf{u}_{K_j} = \frac{\mathbf{Z}_K^\top \mathbf{f}_K}{\| \mathbf{Z}_K^\top \mathbf{f}_K \|_{\mathbf{I}_p}}. \tag{S.9}$$

Vector $\mathbf{u}_{Kj}$, defined the direction of the species j, within the subsets (marginality axis of species j within subset K), for which the average position of species j within subset K is as far as possible from the overall average habitat conditions (G).

In addition, the dispersion or tolerance [noted $T_{m_K}(j)$] of SUs i_s which contains species j, can be calculated. Let $m_{K_{i_s}}$ be the projection of $M_{K_{i_s}}$, onto the marginality axis, as follows:

$$T_{m_K}(j) = \sum_{i_s=1}^{k} f_{K_{i_s/j}} \| G_{K_j} - m_{K_{i_s}} \|^2_{\mathbf{I}_p} \tag{S.10}$$

$T_{m_K}(j)$ represents the subniche breadth of species j under the habitat conditions defined by $\mathbf{Z}_K$. Finally, similarly to the proposal of Dolédec et al. (2000), the projection of the SUs of subset K onto the plane orthogonal to the marginality axis returns a residual tolerance [noted $T_{r_K}(j)$] and the decomposition of the species j total inertia under the subset habitat conditions can be written as follows:

$$I_{T_K}(j) = m_{a_K}(j) + T_{m_K}(j) + T_{r_K}(j) \tag{S.11}$$

The niche variability of the species j thus comprises of the three components advocated by Dolédec et al. (2000): (1) an index of marginality or WitOMIG, *i.e.*, the average distance of species j within subsets to the uniform distribution found in the sampling domain (G); (2) an index of tolerance or subniche breadth and (3) a residual tolerance, *i.e.*, an index that helps to determine the reliability of the subset habitat conditions for the definition of the subniche of species j.

Furthermore the total species inertia $I_T(j)$ calculated in Dolédec et al. (2000), can be recalculated using the inertia of species $I_{T_K}(j)$ as follows:

$$I_T(j) = \sqrt{\sum_{K=1}^{N} (I_{T_K}(j) \times \frac{y_{K.j}}{y_{.j}})^2} \tag{S.12}$$

where $y_{.j}$ corresponds to the total species j abundance in the faunistic table $\mathbf{Y}$.

Subniche parameters calculated from a sub-origin G_K

In the previous section, the subniche parameters are estimated considering the average habitat conditions (G) used by the all species in the assemblage. The subniche parameters can also use the average subset habitat conditions (G_K) and the corresponding subsets of species.

Let us consider again the matrices $\mathbf{Z}_K$, which are centered using their respective mean to yield $\mathbf{Z}_{K^*}$, thus making the global table $\mathbf{Z}^*$:

$$\mathbf{Z}^* = \begin{bmatrix} \mathbf{Z}_{1^*} \\ \vdots \\ \mathbf{Z}_{K^*} \\ \vdots \\ \mathbf{Z}_{N^*} \end{bmatrix} \tag{S.13}$$

Let us consider again the faunistic table, $\mathbf{Fr}_K(k \times t)$ that contains the species frequency (Figure 3). The equations are the same as previously but considering the N centered subset habitat conditions $\mathbf{Z}_{K^*}$ (Details in the Appendix S1). We obtain similarly the total inertia of $\mathbf{Z}_{K^*}$, $I_{T_{K^*}}$, and the inertia of species j, $I_{T_{K^*}}(j)$, can be decomposed into its marginality or WitOMIG_K, $m_{a_{K^*}}(j)$, its tolerance $T_{m_{K^*}}(j)$ and its residual tolerance $T_{r_{K^*}}(j)$.

Let us extract $\mathbf{Z}_{K^*}(k \times p)$, a matrix of $\mathbf{Z}^*(n \times p)$ with k rows. Let the faunistic table, $\mathbf{Fr}_K(k \times t)$ contain the frequency of t species in the k SUs. Let M_{i_s} represent SU i_s of table $\mathbf{Z}_{K^*}(k \times p)$ in the multidimensional space $\mathbb{R}^p$. Let $M_{K^*_{i_s}}$ represent the SU i_s subset habitat conditions of table $\mathbf{Z}_{K^*}$ in the same multidimensional space $\mathbb{R}^p$. The total inertia of the matrix $\mathbf{Z}_{K^*}$, equals:

$$I_{T_{K^*}} = \sum_{i_s=1}^{k} p_{K^*_{i_s}} \parallel M_{K^*_{i_s}} \parallel^2_{\mathbf{I}_p} \tag{S.14}$$

with $p_{K^*_{i_s}}$ being the weight of SU i_s. The inertia of species j considering the matrix $\mathbf{Z}_{K^*}$ equals:

$$I_{T_{K^*}}(j) = \sum_{i_s=1}^{k} f_{K_{i_s/j}} \parallel M_{K^*_{i_s}} \parallel^2_{\mathbf{I}_p} \tag{S.15}$$

The inertia $I_{T_{K^*}}(j)$ represents the inertia weighted by the species profile j. The SUs i_s that do not have species j do not add to the species j inertia. Let us consider a $\mathbf{I}_p$ normed vector $\mathbf{u}_{K^*}$ ($\parallel \mathbf{u}_{K^*} \parallel^2_{\mathbf{I}_p} = 1$). The projection of the k rows of the matrix $\mathbf{Z}_{K^*}$ onto the vector $\mathbf{u}_{K^*}$ results in a vector of coordinates $\mathbf{Z}_{K^*}\mathbf{u}_{K^*}$. Therefore, the average position of species j on $\mathbf{u}_{K^*}$, equivalent to the center of gravity of species j within a subset of habitat conditions is defined as:

$$T_{K^*_j} = \mathbf{f}_K^\top \mathbf{Z}_{K^*}\mathbf{u}_{K^*} \qquad \mathbf{f}_K^\top = (f_{K_{1/j}}, \ldots, f_{K_{i_s/j}}, \ldots, f_{K_{k/j}}). \tag{S.16}$$

With the Eq. S19, marginality within a subset of habitat conditions, or within subset outlying mean index to G_K (WitOMIG_K) of species j [noted $m_{a_{K^*}}(j)$] along $\mathbf{u}_{K^*}$ equals:

$$m_{a_{K^*}}(j) = T^2_{K^*_j} = (\mathbf{f}_K \mid \mathbf{Z}_{K^*}\mathbf{u}_{K^*})^2_{\mathbf{I}_p} = (\mathbf{Z}_{K^*}^\top \mathbf{u}_{K^*} \mid \mathbf{f}_K)^2_{\mathbf{I}_p} \tag{S.17}$$

This marginality represents the deviation between the average position of species j within subsets from the subset habitat origin (G_K). Also equivalent to the distance between the average subset habitat conditions used by species j and the average subset habitat conditions of the subset area.

From Eq. S20, the maximization of $m_{a_{K^*}}(j)$ as for solution $\mathbf{u}_{K^*}$:

$$\mathbf{u}_{K^*_j} = \frac{\mathbf{Z}_{K^*}^\top \mathbf{f}_K}{\parallel \mathbf{Z}_{K^*}^\top \mathbf{f}_K \parallel_{\mathbf{I}_p}}. \tag{S.18}$$

Vector $\mathbf{u}_{K^*j}$, defined the direction of the species j, within the subsets (marginality axis of species j within the subsets), for which the average position of species j within subsets is as far as possible from the subset habitat conditions found in the area G_K.

In addition, the dispersion or tolerance [noted $T_{m_{K^*}}(j)$] of SUs i_s that contains species j can be calculated. Let $m_{K^*_{i_s}}$ be the projection of $M_{K^*_{i_s}}$, onto the marginality axis as follows:

$$T_{m_{K^*}}(j) = \sum_{i_s=1}^{k} f_{K_{i_s/j}} \parallel G_{Kj} - m_{K^*_{i_s}} \parallel^2_{\mathbf{I}_p} \tag{S.19}$$

$T_{m_{K^*}}(j)$ represents the subniche breadth of species j under the subset habitat conditions defined by $\mathbf{Z}_{K^*}$. Similarly to the proposal of (Dolédec et al., 2000), the projection of the k SUs of subset K onto the plane orthogonal to the marginality axis returns a residual tolerance [noted $T_{r_{K^*}}(j)$] and the decomposition of the species j total inertia under the subset habitat conditions equals:

$$I_{T_{K^*}}(j) = m_{a_{K^*}}(j) + T_{m_{K^*}}(j) + T_{r_{K^*}}(j) \tag{S.20}$$

REFERENCES

Dolédec, S., Chessel, D., and Gimaret-Carpentier, C. (2000). Niche separation in community analysis: A new method. *Ecology*, 81(10):2914.
