# Supplementary material for: Within outlying mean indexes: refining the OMI analysis for the realized niche decomposition
Source: PeerJ. 2017 May 23;5:e3364. doi: 10.7717/peerj.3364 (PMC5444369; doi:10.7717/peerj.3364)
Supplement: Supplemental Information 2 — Tables S1 and S2 are the results of the OMI analysis, the value of the WitOMI parameters and their respective P values, for species of both ecological examples. Tables S3 and S4 are the respective code for each species of both ecological examples. [file peerj-05-3364-s002.pdf]

APPENDIX S2: WITOMI RESULTS AND SPECIES' CODE

Table S1. Hydraulic requirement parameters of the 57 invertebrate taxa sampled in spring and autumn. Inertia = total variability; OMI = outlying mean index. P number of random permutations (out of 1000) that yielded a higher value than the observed marginality (OMI, WitOMIG or WitOMIG_K) (the value in **bold** characters are significant, P <0.05). Tol = tolerance, Rtol = residual tolerance. I. = inertia; G (G_k) are the subset marginality WitOMIG (WitOMIG_K) ; $-$ = NA. The WitOMI cannot be calculated when the OMI is not significant (See Discussion for further details). The species is the one use as example for Figure 3D, 3E, and 3F (Species code in Appendix S2; Table S3).

Season	All					Spring					Autumn					Spring					Autumn				
Code	I.	OMI	Tol	Rtol	P	I.	G	Tol	Rtol	P	I.	G	Tol	Rtol	P	I.	G_K	Tol	Rtol	P	I.	G_K	Tol	Rtol	P
AFLU	5.54	1.63	1.16	2.75	**0.05**	5.59	2.46	0.54	2.59	0.00	5.05	4.99	0.04	0.02	0.00	5.89	2.76	0.70	2.43	0.00	5.05	4.99	0.04	0.02	0.00
ANTO	6.26	2.86	0.48	2.92	**0.01**	6.26	2.86	0.48	2.92	0.00	-	-	-	-	-	7.08	3.68	0.41	3.00	0.00	-	-	-	-	-
ATSP	5.26	0.77	1.94	2.54	0.10	-	-	-	-	-	-	-	-	-	-	-	-	-	-	-	-	-	-	-	-
BASP	4.95	0.15	0.92	3.88	**0.00**	5.22	0.37	1.28	3.57	0.01	4.02	0.95	0.86	2.21	0.00	4.89	0.04	2.11	2.74	0.01	4.02	0.95	0.86	2.21	0.00
BIMI	6.44	2.67	1.22	2.56	0.07	-	-	-	-	-	-	-	-	-	-	-	-	-	-	-	-	-	-	-	-
BFAS	5.52	1.63	0.83	3.06	**0.00**	5.52	1.63	0.83	3.06	0.00	-	-	-	-	-	4.86	0.97	1.62	2.27	0.00	-	-	-	-	-
CASP	4.75	2.09	0.70	1.96	**0.00**	3.82	2.28	0.45	1.09	0.00	5.17	2.46	0.75	1.96	0.00	3.98	2.44	0.46	1.08	0.00	5.17	2.46	0.75	1.96	0.00
CESP	5.99	1.36	1.74	2.89	0.14	-	-	-	-	-	-	-	-	-	-	-	-	-	-	-	-	-	-	-	-
CERA	5.24	0.44	1.56	3.24	0.07	-	-	-	-	-	-	-	-	-	-	-	-	-	-	-	-	-	-	-	-
CLEP	5.03	0.44	1.38	3.21	**0.00**	5.04	0.48	0.96	3.60	0.01	4.99	1.70	1.40	1.89	0.00	4.68	0.12	2.26	2.30	0.00	4.99	1.70	1.40	1.89	0.00
CMAR	5.30	2.09	1.10	2.10	**0.00**	5.36	2.17	0.96	2.24	0.00	4.97	3.47	0.60	0.90	0.00	4.80	1.60	1.55	1.65	0.00	4.97	3.47	0.60	0.90	0.00
CHIR	4.44	0.49	1.52	2.42	0.12	-	-	-	-	-	-	-	-	-	-	-	-	-	-	-	-	-	-	-	-
CPIC	5.51	2.83	0.52	2.16	**0.00**	5.51	2.83	0.52	2.16	0.00	-	-	-	-	-	5.72	3.04	0.56	2.12	0.00	-	-	-	-	-
DRSP	4.50	1.07	1.90	1.52	**0.04**	8.57	3.37	2.26	2.94	0.00	3.75	2.11	0.71	0.93	0.00	7.42	2.22	2.13	3.08	0.00	3.75	2.11	0.71	0.93	0.00
DUSP	5.33	0.14	1.74	3.45	**0.05**	5.85	0.59	1.64	3.61	0.00	4.78	1.01	1.72	2.05	0.00	5.38	0.12	3.08	2.18	0.00	4.78	1.01	1.72	2.05	0.00
ECSP	5.35	0.33	1.47	3.55	**0.00**	5.33	0.53	1.48	3.32	0.01	5.49	0.57	1.22	3.70	0.00	4.86	0.06	2.57	2.24	0.01	5.49	0.57	1.22	3.70	0.00
ECTE	5.46	3.09	0.12	2.25	0.12	-	-	-	-	-	-	-	-	-	-	-	-	-	-	-	-	-	-	-	-
ELMA	3.18	0.79	0.04	2.35	0.86	-	-	-	-	-	-	-	-	-	-	-	-	-	-	-	-	-	-	-	-
EPEO	5.68	1.27	0.75	3.66	0.26	-	-	-	-	-	-	-	-	-	-	-	-	-	-	-	-	-	-	-	-
EVIR	5.30	0.34	1.28	3.68	**0.04**	5.30	0.34	1.28	3.68	0.01	-	-	-	-	-	5.20	0.24	2.07	2.89	0.00	-	-	-	-	-
EPAR	269	3.93	0.14	0.80	3.00	0.26	-	-	-	-	-	-	-	-	-	-	-	-	-	-	-	-	-	-	- -
EPYG	4.77	0.14	1.02	3.61	**0.02**	4.77	0.32	0.50	3.95	0.01	4.77	0.47	0.54	3.75	0.00	4.54	0.09	1.10	3.36	0.00	4.77	0.47	0.54	3.75	0.00
ESSP	4.86	0.14	0.86	3.86	**0.00**	5.04	0.65	1.45	2.94	0.00	4.74	0.49	0.47	3.78	0.00	5.00	0.61	1.58	2.81	0.00	4.74	0.49	0.47	3.78	0.00
EGEN	5.05	1.91	0.86	2.28	**0.00**	5.05	1.91	0.86	2.28	0.00	-	-	-	-	-	5.01	1.87	0.91	2.23	0.00	-	-	-	-	-
GASP	5.69	0.36	1.17	4.16	**0.05**	5.87	0.43	1.09	4.35	0.01	2.58	0.62	0.35	1.62	0.00	5.51	0.07	2.19	3.25	0.01	2.58	0.62	0.35	1.62	0.00
HEXO	5.22	0.38	1.25	3.60	**0.00**	5.30	0.44	1.05	3.81	0.01	4.64	2.02	0.82	1.80	0.00	4.92	0.07	2.49	2.37	0.01	4.64	2.02	0.82	1.80	0.00
HPEL	5.24	0.36	1.20	3.69	0.00	5.32	0.45	0.99	3.89	0.01	4.46	2.37	0.30	1.78	0.00	4.93	0.06	2.58	2.30	0.01	4.46	2.37	0.30	1.78	0.00
HYDR	3.57	1.48	0.42	1.67	0.06	-	-	-	-	-	-	-	-	-	-	-	-	-	-	-	-	-	-	-	-
HYSP	5.42	0.34	1.01	4.07	**0.00**	5.44	0.42	0.98	4.05	0.01	5.12	0.76	1.38	2.98	0.00	5.03	0.01	1.85	3.18	0.01	5.12	0.76	1.38	2.98	0.00
HYDS	6.18	2.75	0.83	2.59	**0.03**	6.18	2.75	0.83	2.59	0.00	-	-	-	-	-	7.16	3.73	0.75	2.67	0.00	-	-	-	-	-
LEPT	5.34	2.74	0.47	2.13	0.16	-	-	-	-	-	-	-	-	-	-	-	-	-	-	-	-	-	-	-	-
LESP	4.42	0.31	0.90	3.21	**0.04**	4.61	0.45	1.09	3.07	0.01	2.63	1.24	0.26	1.13	0.00	4.40	0.24	1.31	2.85	0.00	2.63	1.24	0.26	1.13	0.00
LOPAad	4.97	1.20	1.97	1.79	**0.01**	4.97	1.20	1.97	1.79	0.00	-	-	-	-	-	4.87	1.10	2.04	1.73	0.00	-	-	-	-	-
LOPAla	4.57	0.48	1.25	2.85	**0.00**	5.11	0.37	0.47	4.27	0.01	3.72	1.91	0.31	1.50	0.00	4.88	0.14	1.21	3.53	0.01	3.72	1.91	0.31	1.50	0.00
MPOW	5.77	1.21	2.07	2.49	0.27	-	-	-	-	-	-	-	-	-	-	-	-	-	-	-	-	-	-	-	-
MYSA	4.20	3.34	0.28	0.58	0.24	-	-	-	-	-	-	-	-	-	-	-	-	-	-	-	-	-	-	-	-
ORHE	5.58	1.77	0.89	2.92	**0.00**	5.58	1.77	0.89	2.92	0.00	-	-	-	-	-	4.93	1.12	1.73	2.08	0.00	-	-	-	-	-
ONSP	5.18	1.10	1.74	2.34	0.06	-	-	-	-	-	-	-	-	-	-	-	-	-	-	-	-	-	-	-	-
ORTH	5.12	0.23	1.26	3.63	**0.00**	5.39	0.39	0.91	4.09	0.01	4.35	0.90	1.57	1.88	0.00	5.01	0.01	2.50	2.50	0.01	4.35	0.90	1.57	1.88	0.00
ORSP	6.83	1.32	2.33	3.18	0.07	-	-	-	-	-	-	-	-	-	-	-	-	-	-	-	-	-	-	-	-
OTROad	5.32	0.32	0.79	4.21	**0.02**	5.25	0.41	0.98	3.87	0.01	6.23	2.59	1.00	2.64	0.00	5.01	0.16	1.60	3.25	0.01	6.23	2.59	1.00	2.64	0.00
OTROla	5.52	0.23	1.21	4.08	0.06	-	-	-	-	-	-	-	-	-	-	-	-	-	-	-	-	-	-	-	-
PISI	4.43	0.37	1.12	2.94	0.77	-	-	-	-	-	-	-	-	-	-	-	-	-	-	-	-	-	-	-	-
POLY	6.09	1.16	2.02	2.91	0.07	-	-	-	-	-	-	-	-	-	-	-	-	-	-	-	-	-	-	-	-
PBIF	6.95	3.46	0.59	2.90	**0.01**	8.24	7.16	0.33	0.76	0.00	5.87	3.73	0.79	1.36	0.00	9.48	8.39	0.35	0.74	0.00	5.87	3.73	0.79	1.36	0.00
PPUS	5.23	0.14	0.91	4.18	0.15	-	-	-	-	-	-	-	-	-	-	-	-	-	-	-	-	-	-	-	-
RASP	4.76	0.13	0.81	3.82	0.97	-	-	-	-	-	-	-	-	-	-	-	-	-	-	-	-	-	-	-	-
RHIP	6.91	6.12	0.52	0.27	**0.00**	6.91	6.12	0.52	0.27	0.00	-	-	-	-	-	6.36	5.57	0.54	0.25	0.00	-	-	-	-	-
RHYP	5.93	1.93	1.14	2.86	**0.00**	5.93	1.93	1.14	2.86	0.00	-	-	-	-	-	5.28	1.28	1.61	2.39	0.00	-	-	-	-	-
SIGN	5.51	0.53	1.71	3.26	**0.00**	5.51	0.53	1.71	3.26	0.01	-	-	-	-	-	5.30	0.32	2.08	2.90	0.00	-	-	-	-	-
SARG	5.69	0.24	1.27	4.19	**0.02**	6.12	2.03	1.66	2.42	0.00	5.24	0.83	2.00	2.41	0.00	5.44	1.36	1.34	2.74	0.00	5.24	0.83	2.00	2.41	0.00
SIMU	5.34	0.51	1.24	3.59	**0.00**	5.63	0.58	0.94	4.12	0.00	4.38	2.45	0.87	1.06	0.00	5.11	0.06	1.82	3.24	0.01	4.38	2.45	0.87	1.06	0.00
STEN	6.27	0.57	1.59	4.11	0.38	-	-	-	-	-	-	-	-	-	-	-	-	-	-	-	-	-	-	-	-
SCAN	6.36	1.40	1.40	3.56	**0.02**	6.30	3.00	0.45	2.84	0.00	6.47	0.98	0.62	4.87	0.00	6.54	3.25	0.56	2.74	0.00	6.47	0.98	0.62	4.87	0.00
TANYP	4.88	0.73	1.69	2.46	**0.02**	4.88	0.73	1.69	2.46	0.01	-	-	-	-	-	4.95	0.80	1.92	2.23	0.00	-	-	-	-	-
TANYT	5.34	0.17	1.32	3.85	**0.03**	5.48	0.32	0.91	4.25	0.01	4.46	1.41	0.79	2.26	0.00	5.22	0.06	1.52	3.64	0.01	4.46	1.41	0.79	2.26	0.00
TFLU	6.94	2.03	1.56	3.36	0.11	-	-	-	-	-	-	-	-	-	-	-	-	-	-	-	-	-	-	-	-

Table S2. Physical habitat preference parameters of the 12 fish taxa sampled in ten Mediterranean tributaries of the Rhône River. Inertia = total variability; OMI = outlying mean index. P number of random permutations (out of 1000) that yielded a higher value than the observed marginality (OMI, WitOMIG or WitOMIG_K) (the value in **bold** characters are significant, P <0.05). Tol = tolerance, Rtol = residual tolerance. I. = inertia; G (G_k) are the subset marginality WitOMIG (WitOMIG_K). − = NA. The WitOMI cannot be calculated when the OMI is not significant(See Discussion for further details). The species is the one use as example for Figure 4D, 4E, and 4F (Species code in Table S4 in appendix S2).

Altitude	All					Upstream					Downstream					Upstream					Downstream				
Code	I.	OMI	Tol	Rtol	P	I.	G	Tol	Rtol	P	I.	G	Tol	Rtol	P	I.	G_K	Tol	Rtol	P	S.I.	G_K	Tol	Rtol	P
SCU	5.50	2.15	1.50	1.85	0.08	-	-	-	-	-	-	-	-	-	-	-	-	-	-	-	-	-	-	-	-
OTR	5.71	0.08	2.43	3.21	**0.00**	4.74	1.12	1.62	2.01	**0.00**	8.44	4.14	1.09	3.21	**0.00**	3.62	0.00	0.96	2.66	**0.00**	4.47	0.18	1.69	2.60	**0.00**
YTR	5.77	0.26	1.69	3.81	**0.00**	4.92	1.15	1.67	2.10	**0.00**	9.17	5.58	1.59	1.99	**0.00**	3.77	0.00	0.75	3.02	**0.00**	4.98	1.40	1.22	2.37	**0.00**
MIN	5.32	0.45	2.52	2.35	**0.00**	2.82	0.33	0.96	1.54	**0.00**	8.51	4.61	0.81	3.08	**0.00**	2.79	0.30	1.01	1.48	**0.00**	3.93	0.04	0.88	3.01	**0.00**
STO	5.91	0.77	2.41	2.73	**0.00**	2.86	0.20	0.67	1.98	**0.00**	9.28	4.93	0.88	3.48	**0.00**	3.17	0.51	0.78	1.87	**0.00**	4.38	0.03	1.24	3.11	**0.00**
BLA	4.99	0.46	2.27	2.26	**0.00**	2.31	0.20	0.71	1.40	**0.00**	8.61	4.53	0.88	3.19	**0.00**	2.51	0.40	0.73	1.38	**0.00**	4.09	0.02	0.68	3.40	**0.00**
SBA	3.71	0.07	0.51	3.13	0.77	-	-	-	-	-	-	-	-	-	-	-	-	-	-	-	-	-	-	-	-
SON	6.38	4.89	0.75	0.75	**0.03**	-	-	-	-	-	6.38	4.89	0.75	0.75	**0.00**	-	-	-	-	-	2.80	1.31	0.13	1.37	**0.00**
NAS	12.60	10.20	1.62	0.78	**0.00**	-	-	-	-	-	12.60	10.20	1.62	0.78	**0.00**	-	-	-	-	-	4.40	2.00	1.33	1.07	**0.00**
GUD	8.43	4.31	0.95	3.17	**0.00**	1.16	0.25	0.47	0.44	**0.00**	9.47	5.52	0.51	3.44	**0.00**	2.53	1.62	0.02	0.89	**0.00**	4.04	0.09	0.98	2.97	**0.00**
CHU	5.58	1.58	1.69	2.31	**0.00**	1.27	0.03	0.13	1.11	**0.00**	8.61	4.53	0.88	3.19	**0.00**	2.34	1.10	0.28	0.96	**0.00**	4.09	0.02	0.68	3.40	**0.00**
STR	9.92	5.28	1.19	3.46	**0.00**	-	-	-	-	-	11.07	6.86	0.58	3.62	**0.00**	-	-	-	-	-	4.63	0.43	0.84	3.37	**0.00**
BAR	8.34	3.83	1.30	3.21	**0.00**	1.66	0.52	0.07	1.07	**0.00**	9.46	5.26	0.58	3.62	**0.00**	2.60	1.45	0.88	0.27	**0.00**	4.28	0.07	0.60	3.60	**0.00**

Table S3. Invertebrate code from Mérigoux and Dolédec (2004).

Species	Code
Ancylus fluviatilis Müller	AFLU
Antocha sp.	ANTO
Athripsodes sp.	ATSP
Baetis sp.	BASP
Bidessus minutissimus	BIMI
Blephari cera fasciata (Westwood)	BFAS
Caenis sp.	CASP
Ceraclea sp.	CESP
Ceratopogoninae	CERA
Cheumatopsyche lepida (Pictet)	CLEP
Chimarra marginata (Linnaeus)	CMAR
Chironomini	CHIR
Choroterpes picteti Eaton	CPIC
Dryops sp.	DRSP
Dugesia sp.	DUSP
Ecdyonurus sp.	ECSP
Ecnomus tenellus	ECTE
Elmis maugetii (l)	ELMA
Epeorus sp.	EPEO
Ephoron virgo (Olivier)	EVIR
E. parallelepipedus (a)	EPAR
Esolus spp. (l)	ESSP
E. pygmaeus (a) (Ph. Müller)	EPYG
Euleuctra geniculata Stephens	EGEN
Gammarus spp.	GASP
Hydropsyche spp.	HYSP
H. exocellata Dufour	HEXO
H. pellucidula (Curtis)	HPEL
Hydra sp.	HYDR
Hydroptila sp.	HYDS
Leptocerus tineiformis	LEPT
Leuctra sp.	LESP
Limnius opacus (a) Ph. Müller	LOPAad
L. opacus (l) Ph. Müller	LOPAla
Micronecta poweri	MPOW
Mystacides azurea	MYSA
Oligoneuriella rhenana (Imhoff)	ORHE
Onychogomphus sp.	ONSP
Orthocladiinae	ORTH
Orthotrichia sp.	ORSP
Oulimnius troglodytes (a) (Gyllenhal)	OTROad
O. troglotydes (l)	OTROla
Pisidium sp.	PISI
Polycentrus flavomaculatus	POLY
Procloeon bifidum (Bengtsson)	PBIF
Psychomyiia pusilla	PPUS
Radix sp.	RASP
Rhyacophila sp.	RHYP
Rhithrogena sp.	RHIP
Serratella ignita (Poda)	SIGN
Setodes argentipunctellus McLachlan	SARG
Simuliidae	SIMU
Stenelmis canaliculata (a)	STEN
S. canaliculata (l)	SCAN
Tanypodinae	TANYP
Tanytarsini	TANYT
Theodoxus fluviatilis	TFLU

Table S4. Fish code from Dolédec et al. (2000);† Young of the year.

Species	Code
Sculpin (*Cottus gobio*)	SCU
Older trout (*Salmo trutta*)	OTR
Y-O-Y† trout (*Salmo trutta*)	YTR
Minnow (*Phoxinus phoxinus*)	MIN
Stone loach (*Nemacheilus barbatulus*)	STO
Blageon (*Telestes soufia*)	BLA
Southwestern barbel (*Barbus meridionalis*	SBA
Southwestern nase (*Chondrostoma toxostoma*)	SON
Nase (*Chondrostoma nasus*)	NAS
Gudgjeon (*Gobio gobio*)	GUD
Chub (*Leuciscus cephalus*)	CHU
Streambleak (*Alburnoides bipunctatus*)	STR
Barbel (*Barbus barbus*)	BAR

REFERENCES

Dolédec, S., Chessel, D., and Gimaret-Carpentier, C. (2000). Niche separation in community analysis: A new method. *Ecology*, 81(10):2914.

Mérigoux, S. and Dolédec, S. (2004). Hydraulic requirements of stream communities: A case study on invertebrates. *Freshwater Biology*, 49(5):600–613.
